# Evaluation of the Dual Antiviral and Immunomodulatory Effects of *Phallus indusiatus* in a Feline Infectious Peritonitis Model Using PBMCs

**DOI:** 10.3390/vetsci12090847

**Published:** 2025-09-01

**Authors:** Chularat Hlaoperm, Wassamon Moyadee, Emwalee Wongsaengnoi, Wiwat Klankaew, Amonpun Rattanasrisomporn, Atchara Paemanee, Kiattawee Choowongkomon, Oumaporn Rungsuriyawiboon, Jatuporn Rattanasrisomporn

**Affiliations:** 1 Graduate Program in Animal Health and Biomedical Sciences, Faculty of Veterinary Medicine, Kasetsart University, Bangkok 10900, Thailand; chularat.h@ku.th (C.H.); wassamon.moy@ku.th (W.M.); 2 Department of Companion Animal Clinical Sciences, Faculty of Veterinary Medicine, Kasetsart University, Bangkok 10900, Thailand; 3 Department of Veterinary Technology, Faculty of Veterinary Technology, Kasetsart University, Bangkok 10900, Thailand; emwalee.wo@ku.th (E.W.); cvtopr@ku.ac.th (O.R.); 4 Department of Biochemistry, Faculty of Science, Kasetsart University, Bangkok 10900, Thailand; wiwat.klankaew@gmail.com (W.K.); fsciktc@ku.ac.th (K.C.); 5 Interdisciplinary of Genetic Engineering and Bioinformatics, Graduate School, Kasetsart University, Bangkok 10900, Thailand; fgraapr@ku.ac.th; 6 National Omics Center, National Center for Genetic Engineering and Biotechnology (BIOTEC), National Science and Technology Development Agency (NSTDA), Khlong Luang, Pathum Thani 12120, Thailand; atchara.pae@biotec.or.th; 7 Food Biotechnology Research Team, Functional Ingredients and Food Innovation Research Group, National Center for Genetic Engineering and Biotechnology (BIOTEC), National Science and Technology Development Agency (NSTDA), Khlong Luang, Pathum Thani 12120, Thailand

**Keywords:** *Phallus indusiatus*, PBMCs, feline coronavirus (FCoV), feline infectious peritonitis (FIP), FIPV protease, FIPV M^pro^, antiviral, anti-inflammatory

## Abstract

Feline infectious peritonitis (FIP) is a progressive and often fatal disease caused by a virulent biotype of feline coronavirus (FCoV). Although antiviral treatments are now available, relapse and resistance remain ongoing concerns. This study investigates the therapeutic potential of *P. indusiatus*, a medicinal mushroom, for its antiviral and anti-inflammatory activities against FIP. The main protease (FIPV M^pro^) of feline infectious peritonitis virus (FIPV) was recombinantly expressed and purified to facilitate enzyme inhibition screening. *P. indusiatus* exhibited the strongest FIPV M^pro^ inhibitory activity among the 17 mushroom extracts tested (69.2%), showing a notable level of inhibition relative to standard antiviral agents such as lopinavir and ritonavir. To assess its anti-inflammatory potential, PBMCs derived from healthy cats and FIP-associated effusions (FIP fluid) were cultured and stimulated with LPS to induce inflammation. In healthy PBMCs, *P. indusiatus* significantly reduced nitrite levels, with effects similar to dexamethasone. However, PBMCs from FIP fluid, already in an activated state, showed no additional response. Notably, this study is the first to successfully isolate and culture PBMCs from FIP fluid, providing a new platform for future immunological research. These findings suggest that *P. indusiatus* possesses both antiviral and anti-inflammatory properties, positioning it as a potential dual-action therapeutic candidate for FIP. Further investigation into cytokine signaling pathways is warranted to clarify its mechanisms of action and advance future therapeutic development.

## Simple Summary

This study explores the potential of *Phallus indusiatus* (*P. indusiatus*), a medicinal mushroom, as a dual-action treatment for feline infectious peritonitis (FIP), a fatal disease caused by feline coronavirus. *P. indusiatus* demonstrated the most pronounced inhibitory effect among the 17 mushroom extracts tested (69.2% inhibition of FIPV M^pro^), showing a level of activity approaching that of standard antivirals such as lopinavir and ritonavir. The extract also significantly reduced nitric oxide production (a marker of inflammation) in lipopolysaccharide (LPS)-stimulated peripheral blood mononuclear cells (PBMCs) from healthy cats, indicating anti-inflammatory effects. However, immune cells from cats already suffering from FIP, which were in a chronically inflamed state, did not respond to the extract or to dexamethasone. This highlights the complexity of FIP-related inflammation. This study also successfully cultured PBMCs from FIP fluid for the first time, providing a novel in vitro model for future research. Overall, the results suggest that *P. indusiatus* has promising antiviral and anti-inflammatory properties, though further research is needed to isolate active compounds and test in live infection models.

## 1. Introduction

Feline coronavirus (FCoV) is an enveloped, positive-sense single-stranded RNA virus in the Coronaviridae family commonly found in domestic cats [1]. It exists in two biotypes: feline enteric coronavirus (FECV) [2], which typically causes mild gastrointestinal signs and is often self-limiting in immunocompetent cats, and feline infectious peritonitis virus (FIPV), the pathogenic variant responsible for feline infectious peritonitis (FIP) [3]. First reported in 1966, FIP is fatal, immune-mediated disease that primarily affects kittens, adolescents, and young adult cats, and remains a significant cause of mortality in this population [4]. Approximately 12% of FCoV-infected cats progress to FIP, with environmental stress and immune dysfunction as a key contributing factors [5]. Although antiviral drugs have recently become available, delayed diagnosis still results in high mortality, emphasizing the need for timely and accurate detection [6]. Pathogenesis involves efficient replication of FCoV in activated monocytes and macrophages, leading to systemic inflammation, pyogranulomatous lesions, and T-cell depletion [6,7]. Altered cytokine profiles, including elevated interleukin (IL)-1β, IL-6, IL-10, IL-12p40, and tumor necrosis factor-alpha (TNF-α), have been identified in affected cats, indicating that immune dysregulation plays a key role in the advancement of FIP [8].

Edible mushrooms have been reported to possess multiple biomedical properties, including antiviral, anti-inflammatory activities, and immunomodulatory effects through cytokine stimulation [7,8]. These effects are largely attributed to bioactive compounds such as polysaccharides, terpenoids, and alkaloids. These compounds enhance phagocytic activity, increase IL-1β and TNF-α expression, and activate key signaling pathways such as ERK1/2, JNK, and p38 MAPKs. They also promote NF-κB p65 phosphorylation and nuclear translocation and can bind to macrophages via Toll-like receptor 4 (TLR4) [8]. Certain mushrooms may also help alleviate clinical signs associated with cytokine storms. While some compounds have shown antiviral activity, scientific evaluation in feline models remains limited.

**Figure 1:**
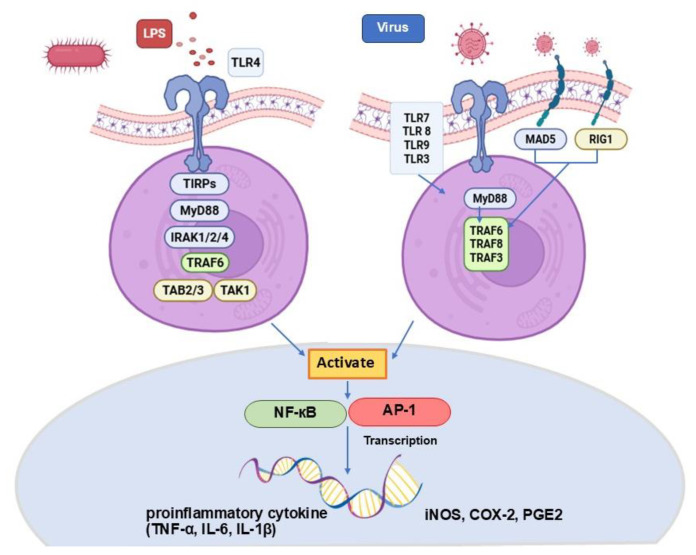
This figure illustrates the activation of TLR–NF-κB and AP-1 signaling pathways in response to LPS or viral stimulation, resulting in the upregulation of inducible iNOS, COX-2, PGE2, and pro-inflammatory cytokines. This schematic was adapted from Luo R. et al., 2025, Yu, L. et al., 2024, and Hennessy & McKernan., 2021 [9,10,11].

In the preliminary inflammation model of this study, both lipopolysaccharide (LPS) and FIPV (or viruses in general) are potent activators of the NF-κB and AP-1 signaling pathways [9,10,11]. These pathways function as central molecular “switches” that regulate the transcription of various inflammatory mediators, including NO, inducible nitric oxide synthase (iNOS), cyclooxygenase-2 (COX-2), and prostaglandin E2 (PGE2), and the pro-inflammatory cytokines (e.g., TNF-α, IL-6, and IL-1β) [12,13]. Therefore, using LPS as an inflammatory stimulant in PBMCs to investigate the anti-inflammatory effects of mushroom extracts is well justified as a preliminary inflammation model, as it activates shared pathways involved in cytokine and iNOS expression mechanisms that are also triggered by FIPV (Figure 1).

Coronaviruses are RNA viruses that hijack the host’s translational machinery to generate viral proteins. The resulting polyproteins must be cleaved to release individual functional proteins necessary for viral replication and transcription. The virus encodes proteases, including the main protease (M^pro^), also known as 3CLpro, which has conserved sequences that facilitate the assembly of the viral replicase complex [14]. The M^pro^ protein of FCoV is essential for the replication cycle, as observed in other members of the Coronaviridae family [15]. Therefore, FIPV M^pro^ protein is considered a prominent drug target for antiviral therapy against FIP (e.g., GC376) [14,16].

The immune system plays a crucial role in controlling viral infections, including the suppression of FCoV replication and prevention of progression to FIP. In this study, 17 mushroom extracts were screened as potential candidates for alternative immunomodulatory strategies in cats. Screening was conducted to assess antiviral activity by them against FIPV M^pro^ to identify active compounds. The extract with the strongest inhibitory effect was selected for further assessment of its anti-inflammatory potential. PBMCs were isolated from both healthy cats and cats with FIP-associated effusions, including pleural, peritoneal, or a combination of both, and were cultured under optimized conditions. These PBMCs were then stimulated with LPS and treated with the selected extract to assess nitrite-scavenging activity. The resulting data will serve as a foundation for future investigations into cytokine modulation, particularly in the context of FIP-related inflammation and antiviral response.

## 2. Materials and Methods

### 2.1. Ethics Statement

This study was approved by the Institutional Animal Care and Use Committee of Kasetsart University, Bangkok, Thailand, under protocol number ACKU67-VET-124. Additionally, all sample collections were performed with the informed consent of the cat owners.

### 2.2. Protein Expression and Purification

The expression and purification of FIPV’s main protease in this study, called “FIPV M^pro^”, have been described previously [17]. In this study, the FIPV M^pro^ was derived from the FIPV WSU-79-1146 strain. The coding sequence for M^pro^ included an N-terminal 10xHis tag and a TEV cleavage site, resulting in a recombinant protein with an approximate molecular weight of 37.2 kDa. The gene was synthesized into the pGEX-4T-1 vector (GenScript, USA) and transformed into *Escherichia coli* strain BL21(DE3) for protein expression. Cultures were grown in LB medium incubated at 37 °C for 3 to 4 h, the absorbance was measured at OD 600 to 0.8, and then the sample was induced by 0.4 mM isopropyl-β-d-thiogalactopyranoside at 37 °C for 18–20 h. The FIPV M^pro^ was purified by Ni^2+^ affinity column (Cytiva AB, Sweden). Western blotting was performed to confirm the protein expression, using an anti-His tag primary antibody (Thermo Fisher Scientific, USA) and an HRP-conjugated Goat Anti-Mouse IgG secondary antibody (Thermo Fisher Scientific, USA) for signal detection.

### 2.3. Inhibition of FIPV M^pro^ Protease

The FIPV M^pro^ modified with Dabcyl-KTSAVLQSGFRKM-E (Edans) (Genscript USA, Piscataway, NJ, USA) was prepared in a buffer containing 20 mM Tris (pH 7.5), 100 mM NaCl, 2 mM DTT, and 0.05 mM EDTA. The enzyme activity was subsequently examined in a 384-well plate containing 1 µM FIPV M^pro^ in buffer solution. Subsequently, enzyme activity inhibitors, including lopinavir and itonavir as positive controls, were added for comparison with 10 µg/mL mushroom extracts. The final inhibitor concentration in the reaction was set to 10 µg/mL. The reaction was initiated by adding the substrate at a final concentration of 40 µM. The increase in fluorescence intensity of the substrate at 340 nm was measured to determine the relationship between Relative Fluorescence Units (RFUs) per second, using a microplate reader (Infinite 200, Tecan Group Ltd., Männedorf, Switzerland) and GraphPad version 8.0 software, as described in Equation (1).

(1) $$\%\;\mathrm{Relative inhibition}=V_{0}\;enz-\left(\frac{V_{0}\;Inhibitor\times 100}{V_{0}\;enz}\right)$$

V_0_ represents the initial reaction velocity of the enzyme. It indicates how fast the enzyme converts substrate to product at the start of the reaction. V_0_ enz is the initial reaction velocity of the enzyme without an inhibitor. V_0_ Inhibitor is the initial reaction velocity of the enzyme in the presence of an inhibitor.

Given the constraints associated with cell culture systems and the limited availability of animal-derived samples, particularly those obtained from experimental models, this phase of the study was designed to reduce animal usage by utilizing an in vitro screening approach. This model was employed to identify mushroom extracts exhibiting the highest inhibitory activity against the FIPV M^pro^. The extract demonstrating the most potent inhibitory effect will be selected for further investigation of its anti-inflammatory properties.

### 2.4. Animals and Experimental Design

This research was approved for sample collection from the Kasetsart University Veterinary Teaching Hospital in Bangkok, Thailand. PBMCs were isolated from healthy donor cats (*n* = 20). In parallel, pleural and/or ascitic fluid samples were collected from cats clinically diagnosed with effusive FIP, based on a combination of consistent clinical, laboratory, and imaging findings. These included serum biochemical and hematological abnormalities, clinical signs such as anorexia, depression, intermittent fever, abdominal distension, or dyspnea due to pleural effusion. Radiographic or ultrasonographic imaging confirmed the presence of fluid accumulation in body cavities and involvement of internal organs. Fluid analysis and cytology revealed pyogranulomatous inflammation, with a proteinaceous, granular background, and a cell population consisting of macrophages, non-degenerate neutrophils, and few lymphocytes. The effusions were classified as modified transudates or non-septic exudates. The diagnosis was further supported by additional findings, including a serum albumin-to-globulin (A:G) ratio of less than 0.8, a positive Rivalta’s test, and/or a positive PCR result for FCoV, in accordance with previously published criteria [18,19]. The pleural and/or ascitic fluid samples were subsequently used for mononuclear cell isolation. These samples (*n* = 20) were subsequently used for mononuclear cell isolation. For simplicity, mononuclear cells isolated from effusions are referred to as “PBMCs from FIP fluid” throughout this study. The sample size was derived from data in other similar studies. All analyses were performed using GraphPad Prism version 9 (GraphPad Software, LLC, Boston, MA, USA) [20].

### 2.5. The Preparation of the Mushroom Extraction Procedure

A total of 17 types of medical mushrooms were selected, including *Pleurotus ostreatus*, *Flammulina filiformis*, *Agrocybe cylindracea*, *Lentinula edodes*, *Tuber Uncinatum*, *Lignosus rhinocerotis*, *Volvariella volvacea*, *Ganoderma oregonense*, *Ganoderma formosanum*, *Ganoderma lucidum*, *Inonotus obliquus*, *Hericium erinaceus*, *Psilocybe cubensis*, *Phellinus igniarius*, *Dasoclema simensis*, *Pleurotus ostreatus,* and *Phallus indusiatus*. A dry sample of 100 g was air-dried and grinded by mixer grinder, immersed in 95% ethyl alcohol (ethanol) 200 mL at a temperature of 37 °C, and shaken for 12 h. Afterward, the liquid part was separated and the ethanol was evaporated using a rotary evaporator under vacuum. The crude extract was stored at a temperature of −20 °C until analysis. The extracts from the 17 types of mushrooms will be further tested for their FIPV M^pro^ inhibitory activity. In this study, crude extracts were prepared using the same stock and extraction protocol described by Theeraraksakul, K. et al., 2023 [21].

### 2.6. Preparation of PBMCs

Whole blood from healthy donor cats and effusion samples (pleural, ascitic, or both) from FIP cats were processed to isolate PBMCs by separating the buffy coats using centrifugation with Lymphoprep™ (Corning^®^, USA) at 600 g for 30 min to isolate PBMCs. Subsequently, complete RPMI-1640 medium (Corning^®^, USA) containing 10% fetal bovine serum (FBS; Gibco^®^, USA) and 1% penicillin–streptomycin (Gibco^®^, USA) was added, and the cell concentration was adjusted to 1 × 10^6^ cells/mL. The cells were counted, and their viability was assessed by mixing the separated cells with a 0.4% Trypan Blue solution (Gibco^®^, USA) in a 1:1 ratio. A hemocytometer was used to count the cells under a microscope.

In the case of isolating PBMCs from effusion samples, referred to in this study as “PBMCs from FIP fluid” the sample characteristics of the FIP fluid were carefully considered. If the sample exhibited high viscosity, it was diluted with 1× phosphate-buffered saline (PBS; Cytiva HyClone™, USA) to facilitate efficient cell pelleting. The diluted sample was then centrifuged at 600× *g* for 10 min. The supernatant was discarded, and the cell pellet was retained. The pellet was subsequently washed three times with RPMI medium at a 1:1 ratio, each wash involving centrifugation at 600× *g* for 10 min. Once the cells were isolated, cell counting was performed, and the cells were cultured under the same conditions as previously described.

### 2.7. In Vitro Optimize Toxicity of P. indusiatus, LPS, and DMSO

The toxicity of *P. indusiatus* extract at concentrations of 10,000, 5000, 2500, 625, 156, 78, 39, and 19 µg/mL was tested on PBMCs isolated from the whole blood of donor cats. PBMCs (1 × 10^6^ cells/mL) were seeded into a 96-well plate and pre-incubated for 1–2 h in a humidified atmosphere with 5% CO_2_ at 37 °C. Subsequently, the cells were treated with various concentrations of *P. indusiatus* extract dissolved in 0.5% DMSO. The PBMCs were incubated for 24 h. Following treatment, cell viability was assessed using the Cell Counting Kit-8 (CCK-8) colorimetric assay (Dojindo, Kumamoto, Japan). Briefly, 10 µL of CCK-8 solution was added to each well and incubated for 2–4 h. The absorbance of each well was then measured at 450 nm using a microplate reader (EnSight^®^ Multimode Plate Reader, PerkinElmer, Waltham, MA, USA). Cell viability was determined by calculating the percentage of absorbance relative to the control group (non-treated PBMCs).

The testing involved determining optimal concentrations and exposure times in generating PBMCs after stimulation with LPS (Sigma-Aldrich^®^, Israel) at various concentrations (0.001, 0.01, 0.1, and 1 µg/mL). Additionally, testing was conducted to determine suitable concentrations for cell viability using dimethyl sulfoxide (DMSO, Uvasol, Japan) at concentrations of 0.5%, 1%, 1.5%, and 2%, compared to a control group without DMSO. Cell viability was assessed using the CCK-8 colorimetric assay (Dojindo, Kumamoto, Japan) under the same conditions as described previously.

### 2.8. Anti-Inflammatory Assay Using Nitrite Scavenging Activity

To determine the effect of *P. indusiatus* extract on nitrite production, a primary breakdown product of nitric oxide, cells were suspended in a 96-well plate containing complete RPMI-1640 medium and pre-incubated for 1–2 h in a humidified atmosphere with 5% CO_2_ at 37 °C. Subsequently, the cells were treated with *P. indusiatus* at a concentration of 19 µg/mL and dexamethasone at 5 µg/mL as a positive drug control in the presence of LPS (1 µg/mL) and incubated for 24 h. Nitrite production was determined by calculating the percentage of absorbance relative to the control group (non-treated PBMCs) using the Griess Reagent System (Promega, USA). A parallel test was conducted on PBMCs isolated from FIP fluid under the same conditions, but without LPS stimulation. This study investigates the potential effects of *P. indusiatus* extract on inflammation in PBMCs isolated from whole blood and fluid samples of cats diagnosed with FIP. The primary aim was to evaluate whether the extract can modulate inflammation by measuring nitrite concentrations, which is a marker of inflammatory response.

### 2.9. Statistical Analysis

Data are presented as mean ± Standard Error of the Mean (SEM), with each experiment replicated at least twice to ensure reliability. PBMC viability from whole blood and FIP fluid was compared using two-way ANOVA, with comparisons made between cell means within each row (*p* < 0.05, *n* = 5). The effect of varying LPS concentrations (0.001, 0.01, 0.1, and 1 µg/mL) on nitrite levels was assessed after 24 h using one-way ANOVA, compared to a no-LPS control (*p* < 0.05). The impact of 19 µg/mL *P. indusiatus* and 5 µg/mL dexamethasone on nitrite production in PBMCs from donor cat whole blood was evaluated after 24 h, with data shown as mean ± SEM (*n* = 8), comparing column means. Inflammation modulation in PBMCs from FIP fluid treated with *P. indusiatus* extract (*n* = 5) was analyzed using one-way ANOVA (*p* < 0.05). Differences in treatment responses between PBMCs from whole blood and FIP fluid were assessed by two-way ANOVA (*p* < 0.05, *n* = 4).

## 3. Results

### 3.1. Expression and Purification of FIPV M^pro^

**Figure 2:**
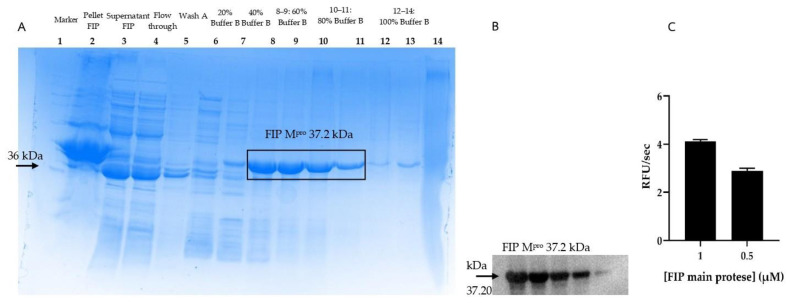
Protein purification was performed using a Ni^2+^ affinity column, with protein expression induced by 0.4 mM IPTG. The FIPV M^pro^ was detected at 37.2 kDa on an SDS-PAGE gel. lane1: Marker, lane2: Pellet FIP, lane3: Supernatant FIP, lane4: Flowthrough, lane5: Wash A, lane6: 20% Buffer B, lane7: 40% Buffer B, lane 8–9: 60% Buffer B, lane10–11: 80% Buffer B, lane12–14: 100% Buffer B (**A**). Western blot analysis confirmed the presence of the FIPV M^pro^ protein at 37.20 kDa (**B**). The reaction rates of the FIPV M^pro^ enzyme (Relative Fluorescence Units/sec) at enzyme concentrations of 1 and 0.5 µM (**C**).

Recombinant His-tagged FIPV 3CLpro, expressed in *E. coli* BL21 (DE3), was purified using a Ni^2+^ affinity column. The expression of the FIPV M^pro^ or FIPV-3C-like Protease, (3CL^pro^), was induced with 0.4 mM isopropyl β-d-1-thiogalactopyranoside (IPTG), followed by incubation for 18–24 h. Quantitative analysis using SDS-PAGE confirmed the expression of the FIPV M^pro^ protein with a molecular weight of 37.20 kDa (Figure 2A).

Qualitative analysis was performed using the Western blot technique, which utilized the specificity of the primary antibody (Anti-His Tag (HRP)) for proteins labeled with a 6x His-Tag. A secondary antibody (Goat Anti-Mouse IgG (HRP)) was employed to bind with HRP substrates. This revealed a band corresponding to the FIPV M^pro^ protein at a size of 37.20 kDa (Figure 2B). The results of determining the optimal concentration of FIPV M^pro^ enzymatic activity, using Dabcyl-KTSAVLQSGFRKM-E (Edans) as the substrate at a final concentration of 40 µM, showed that the reaction rate (or fluorescence intensity) of the substrate increased as the enzyme concentration was raised to 0.5 and 1 µM, separately (Figure 2C).

### 3.2. Inhibition of FIPV M^pro^ Activity by Antiviral Drugs and Crude Mushroom Extract

**Figure 3:**
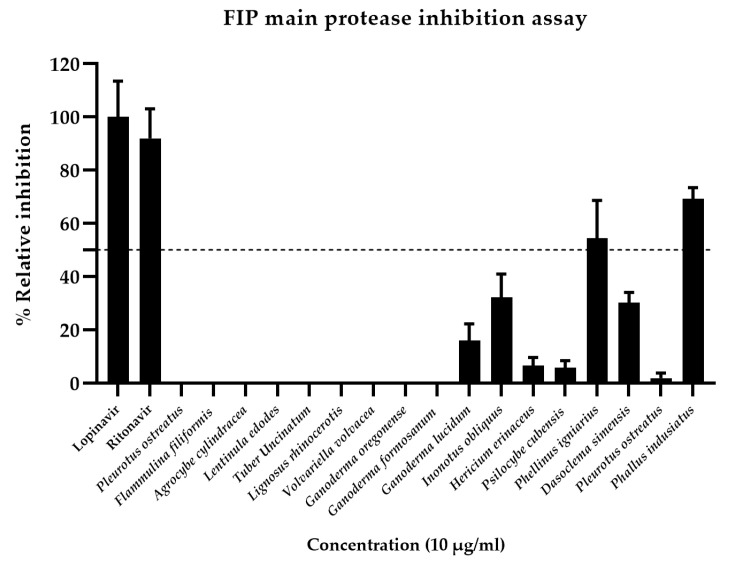
The relationship between the percentage inhibition of FIPV M^pro^ (1 µM) and crude mushroom extracts at a concentration of 10 µg/mL was evaluated. The inhibition of FIPV M^pro^ enzyme activity was evaluated at a concentration of 1 µM. At a concentration of 10 µg/mL, lopinavir inhibited enzyme activity by 84.13%, while ritonavir inhibited it by 71.88%. *P. indusiatus* exhibited the highest inhibition of FIPV M^pro^ activity at 69.2%. The dotted line represents 50% relative inhibition.

The effect of various inhibitors on FIPV M^pro^ activity at a concentration of 1 µM was evaluated using protease inhibitors or antiviral drugs, with lopinavir and ritonavir (National Cancer Institute (NCI, USA)) serving as positive controls for comparison with mushroom extracts. Lopinavir and ritonavir were used at final concentrations of 10 and 100 µg/mL to assess enzyme inhibition by monitoring the reaction rate. This was performed by measuring the fluorescence intensity over 30 min. The initial reaction rate (V_0_) was then used to calculate the percentage of enzyme activity inhibition. At a concentration of 10 µg/mL, lopinavir inhibited enzyme activity by 84.13%, while ritonavir inhibited it by 71.88% (Figure 3).

Relative inhibition of the FIPV M^pro^ assay was evaluated using crude mushroom extracts. The experimental results showed that *P. indusiatus* exhibited the highest inhibition of FIPV M^pro^ activity at 69.2%. Therefore, the extract from *P. indusiatus* was selected for further testing on its anti-inflammatory effects (Figure 3).

### 3.3. Optimization of PBMC Culture Conditions Using Whole Blood Samples and FIP Fluid

**Figure 4:**
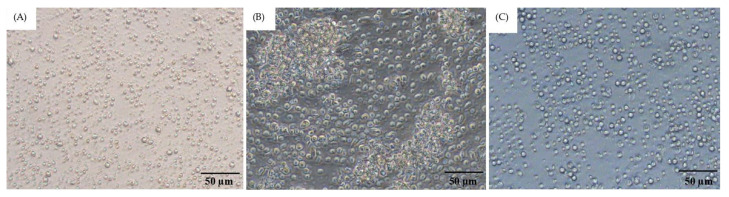
Morphological studies by inverted microscope at actual magnification 50 µm on PBMCs. Normal PBMCs without clumping can be observed when isolated from whole blood (**A**). PBMCs isolated from the fluid showed signs of platelet clumping (**B**). PBMCs separated from FIP fluid without clumping are shown in (**C**).

The characteristics of PBMCs isolated from both whole blood of donor cat and FIP fluid revealed that PBMCs from whole blood contained a small number of platelets and red blood cell contamination (Figure 4A). In contrast, PBMCs isolated from FIP fluid were less pure in terms of white blood cells compared to those from whole blood. The PBMCs sometimes exhibited platelet clumping, which was attributed to the diseased state of the animals. Occasionally, proteins were also present, contributing to the platelet clumping (Figure 4B). Therefore, for the PBMCs isolated from FIP fluid, samples without platelet clumping will be used (Figure 4C).

The experimental results show that at a DMSO concentration of 0.5%, the viability of PBMCs (1 × 10^6^ cells/mL) isolated from whole blood and FIP fluid was 93.75 ± 2.92% and 90.23 ± 2.1%, respectively. This concentration is suitable for further use as a solvent for extracts as it is non-toxic to the cells. However, concentrations of 1%, 1.5%, and 2% were found to be unsuitable as solvents since the viability of PBMCs was below 90%. Therefore, in this study, DMSO at a concentration not exceeding 0.5% was chosen as the solvent for the crude mushroom extract, because PBMCs isolated from both whole blood and FIP fluid had a viability percentage greater than 90% (shown in Figure S1)

The viability assessment of PBMCs (1 × 10^6^ cells/mL) isolated from whole blood and FIP fluid, conducted using the Trypan Blue Exclusion Assay, revealed that on Day 0, cell viability exceeded 90% for both sources, making them suitable for experiments. By Day 1, PBMCs from whole blood remained suitable for experimentation, while PBMCs from FIP fluid were not, as their viability had dropped below 90%. When comparing each day to Day 0, no statistically significant differences were observed from Day 1 to Day 3, whereas statistically significant differences were observed from Day 4 to Day 5.

**Figure 5:**
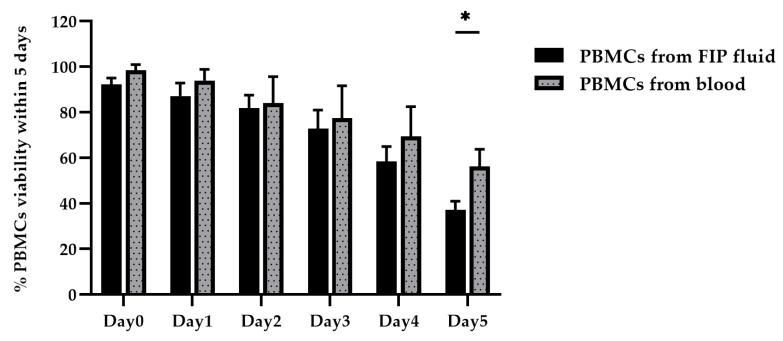
The PBMC viability was analyzed by comparing PBMCs from whole blood collection and FIP fluid samples using a two-way ANOVA. The data are presented as mean ± SEM for PBMCs viability, comparing each cell mean with the other cell means in the same row (*n* = 5). * *p* < 0.05, PBMCs from FIP fluid vs. PBMCs from blood.

A comparison of PBMC viability isolated from both whole blood and FIP fluid was performed using a two-way ANOVA (*p* < 0.05). The results indicated no statistically significant differences in PBMC viability between the two sample types from Day 0 to Day 4. However, on Day 5, a statistically significant difference in PBMC viability was observed between PBMCs isolated from whole blood and FIP fluid (Figure 5).

However, in this study, PBMCs isolated from samples will be used for testing within 24 h, as they exhibit the highest cell viability, exceeding 90% [22]. This summary confirms that PBMCs isolated from both donor and fluid of FIP retain high viability and are appropriate for subsequent experimental applications.

### 3.4. LPS-Stimulated PBMCs

**Figure 6:**
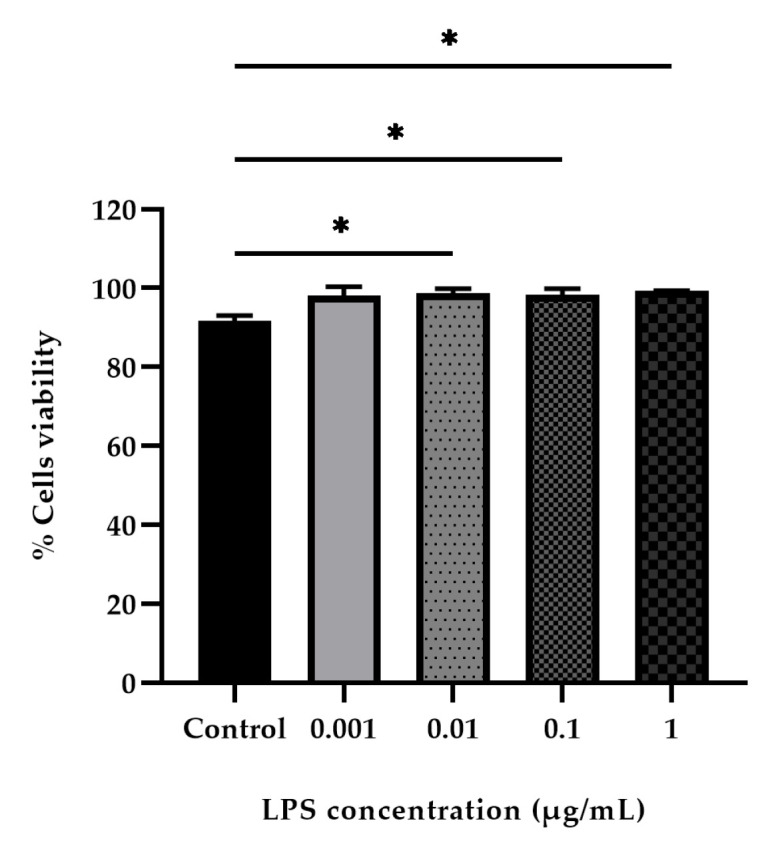
Testing the appropriate concentration of LPS compared to the control group without adding LPS, using concentrations of 0.001, 0.01, 0.1, and 1 µg/mL for 24 h, which were analyzed by one-way ANOVA; * *p* < 0.05 revealed statistically significant differences (*n* = 3).

PBMCs isolated from donor cat blood samples were stimulated with LPS at concentrations ranging from 1 to 0.001 µg/mL for 24 h. Cell viability and proliferation were then assessed using the CCK-8 colorimetric assay. The results showed that increasing LPS concentrations significantly enhanced cell viability. In the control group without LPS stimulation, cell viability was 91.60 ± 0.85%, whereas at the highest LPS concentration (1 µg/mL), cell viability increased to 99.21 ± 0.10% (Figure 6).

Subsequently, a comparison of PBMCs viability at each concentration with the control group without LPS addition revealed that at a concentration of 0.001 µg/mL, there was no statistically significant difference compared to the control group. However, at concentrations of 0.01, 0.1, and 1 µg/mL, there were statistically significant differences from the control group (Figure 6). The concentrations of LPS used did not exhibit cytotoxicity, as cell viability exceeded 90% in all cases. Therefore, an LPS concentration of 1 µg/mL was selected to stimulate its effect on inducing cellular inflammation.

### 3.5. Effect of P. indusiatus on the Cell Viability of PBMCs

The toxicity test of *P. indusiatus* extract at various concentrations on PBMCs derived from the whole blood of donor cats over 24 h was assessed using the CCK-8 colorimetric assay. The results revealed that at concentrations of 10,000, 5000, 2500, 625, 156, 78, and 39 µg/mL, the extract remained toxic to the cells, as cell viability was below 90%. The PBMCs exhibited morphological changes, such as reduced size and loss of sheen, indicating cell death, which was distinct from the control group. However, at a concentration of 19 µg/mL, the cells retained their normal morphology, showing no changes compared to the control group, which is consistent with the 90% viability results. Consequently, a concentration of 19 µg/mL is suitable for further testing. The IC_50_ value was calculated to be 1890 µg/mL (as shown in Table S1).

### 3.6. Evaluation of Nitrite Scavenging Activity of P. indusiatus Extract in LPS-Stimulated PBMCs

**Figure 7:**
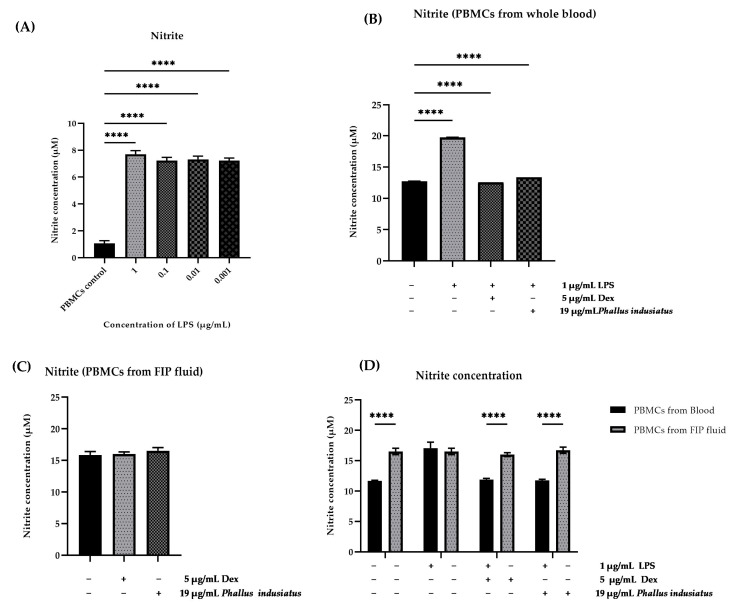
The effect of LPS on nitrite production in stimulated PBMCs was quantified after 24 h of exposure to LPS at concentrations of 0.001, 0.01, 0.1, and 1 µg/mL (**A**). The effect of 19 µg/mL *P. indusiatus* and 5 µg/mL dexamethasone (Dex) on nitrite production in stimulated PBMCs isolated from whole blood of donor cats was quantified after 24 h. The data are presented as mean ± SEM, comparing the mean of each column with the means of every other column (*n* = 8) (**B**). The stimulation and suppression of inflammation in PBMCs isolated from FIP fluid using *P. indusiatus* extract (*n* = 5) (**C**). There was analysis by one-way ANOVA; *p* < 0.05 reveals statistically significant differences. The difference in response to treatments between PBMCs isolated from whole blood and those from FIP fluid was analyzed using two-way ANOVA (*p* < 0.05) (*n* = 4) (**D**). **** *p* < 0.0001.

The evaluation of nitrite (NO_2_^−^) concentration, resulting from the oxidation of nitric oxide (NO), which serves as an indicator for assessing inflammation in LPS-stimulated cells, revealed statistically significant differences compared to the control group at LPS concentrations of 1, 0.1, 0.01, and 0.001 µg/mL (Figure 7A). The nitrite production measured showed a concentration of 7.7 µM, indicating the induction of inflammation in PBMC stimulated with LPS at the respective concentrations. However, considering the viability of PBMCs, it was found that at a concentration of 1 µg/mL, the cells exhibited the highest viability, consistent with the cell viability results assessed using the CCK-8 assay, which showed 99.21 ± 0.01% (Figure 7A). Therefore, an LPS concentration of 1 µg/mL was selected for inducing inflammation in PBMCs.

The test results showed that PBMCs stimulated with 1 µg/mL LPS (negative control) released a nitrite concentration of 19.73 µM. In contrast, the group treated with the anti-inflammatory drug dexamethasone at 5 µg/mL (positive control) exhibited a reduced nitrite concentration of 12.56 µM. Moreover, the group treated with 19 µg/mL of *P. indusiatus* mushroom extract demonstrated a further reduction in nitrite concentration to 13.36 µM. These results suggest that the *P. indusiatus* mushroom extract may have potential anti-inflammatory properties (Figure 7B).

The results also showed that PBMCs isolated from FIP fluid exhibited inflammation, as assessed by the nitrite concentration shown in Figure 7C, which measured 15.87 µM without stimulation by LPS. This indicates that the cat with FIP already had inflammation in its PBMCs. In the group treated with the anti-inflammatory drug dexamethasone (positive control), the nitrite concentration was 16.13 µM, which was not statistically different from the control group. Similarly, the test group treated with *P. indusiatus* extract had a nitrite concentration of 16.52 µM, which also showed no significant difference compared to the control group.

Figure 7D shows a statistically significant difference between PBMCs isolated from blood and those from FIP fluid. PBMCs from FIP fluid exhibited a higher level of cellular inflammation compared to those from blood. However, when comparing PBMCs isolated from blood and stimulated with LPS to PBMCs from FIP fluid, no significant difference was found (Figure 7D). This statistical analysis confirms that PBMCs isolated from FIP fluid are true inflammatory responses. These results indicate that, since there is no significant difference between PBMCs isolated from blood stimulated with LPS and PBMCs from FIP fluid, the latter are naturally inflamed [23]. In the experimental groups treated with dexamethasone (positive control) and the extract of *P. indusiatus*, a statistically significant difference was observed. PBMCs isolated from blood showed a reduction in nitrite production in both the positive control group and the group treated with *P. indusiatus* extract. However, PBMCs isolated from FIP fluid did not exhibit a reduction in nitrite production.

### 3.7. Phytochemical Composition and Literature Support for Anti-Inflammatory Activity

In this study, a crude extract of *P. indusiatus* was prepared following the extraction protocol described by Theeraraksakul, K. et al., 2023 [21]. Phytochemical analysis confirmed the presence of key anti-inflammatory constituents, including phenolic compounds, flavonoids, and tannins. The extract demonstrated inhibitory effects on the production of nitric oxide (NO), interleukin-1 (IL-1), interleukin-6 (IL-6), and tumor necrosis factor-alpha (TNF-α). Furthermore, their study reported that the crude extract of *P. indusiatus* also contained various bioactive compounds, such as catechins, flavonoids, polysaccharides, monosaccharides, mucopolysaccharides, allantoin, alkaloids, epicatechin, gallic acid, caffeic acid, and other polyphenolic compounds are also important contributors to the anti-inflammatory activity of the extract [24,25,26]. Although the crude extract of *P. indusiatus* has not been previously evaluated for its anti-inflammatory effects in cat PBMCs, previous studies have identified bioactive compounds within the extract that possess anti-inflammatory properties. In this study, the observed reduction in NO production in LPS-stimulated PBMCs isolated from healthy cats serves as an important in vitro model (Figure 7B). These findings provide a basis for the future development of anti-inflammatory strategies targeting monocytes and macrophages infected with the FIP virus (Table 1).

**Table 1:** Pharmacological results of *P. indusiatus* efficacy from in vitro studies.

Preparation	Bioactive Compound	Experimental Model	Key Findings	References
Crude extract, hot water, and ethanol.	Phenolics, flavonoids, and tannins.	Murine macrophages used to study the anti-inflammatory effects of LPS stimulation.	The anti-inflammatory activity was demonstrated by a reduction in the level of released nitric oxide (NO).	Theeraraksakul, K. et al., 2023 [21]
Crude extract, peel and green mixture, core, and whole mushroom.	Catechins, flavonoids, polysaccharides, monosaccharides, mucopolysaccharides, allantoin, alkaloids, polyphenolic compounds, epicatechin, gallic acid, and caffeic acid.	The experiment was conducted using LPS-induced RAW 264.7 macrophages.	The anti-inflammatory activity was demonstrated by the inhibition of NO, IL-1β, IL-6, and TNF-α secretion.	Ruksiriwanich, W. et al., 2022., Nazir, Y., et al. 2021., Sedtananun, S. et al., 2025 [24,25,26]
Hot water extract of crude polysaccharide from mushroom fruiting body.	Polysaccharides.	Colitis was induced in mice using dextran sulfate sodium (DSS).	Reduced the expression of inducible iNOS	Kanwal, S. et al., 2020 [27]
This study utilized an ethanolic extract derived from the whole mushroom.	Phenolics, flavonoids, and tannins. [21]	PBMCs from healthy cats were stimulated with LPS and PBMCs from FIP fluid	The anti-inflammatory activity was demonstrated by the inhibition of NO in PBMCs isolated from healthy cats.	Hlaoperm, C. et al.

Bioactive compounds from *P. indusiatus* demonstrate biochemical and cellular mechanisms of action, ranging from general antioxidant and anti-inflammatory effects to the modulation of specific signaling pathways [7]. Therefore, based on the findings of this study and prior research, there is compelling evidence that extracts from *P. indusiatus* possess anti-inflammatory properties and may modulate the immune response, potentially contributing to the inhibition of FIPV in cats.

## 4. Discussion

In this study, the FIPV M^pro^ had a size of 34 kDa, which increased to 37.2 kDa after the addition of a 10 × His tag and a TEV cleavage site at the N-terminus. The FIPV M^pro^ protein was induced by IPTG and analyzed both quantitatively and qualitatively (Figure 2A). Notably, the FIPV M^pro^ comparable in size to the SARS-CoV-2 M^pro^, which has a molecular weight of approximately 33 kDa [28]. These findings indicate that the research was successful at this stage.

The present study revealed a significant finding: the mushroom extract demonstrated FIPV protease inhibition at 69.2%, indicating its potential as a natural source of antiviral compounds. This activity is slightly lower than both ritonavir (71.88%) and lopinavir (84.13%), both of which are established broad-spectrum protease inhibitors clinically used in Human Immunodeficiency Virus (HIV) therapy [29] (Figure 3). These agents have also been shown to inhibit SARS-CoV by strongly binding to its M^pro^ [30,31]. In our study, they served as general protease inhibitory benchmarks, since they are not used for treating FIP. It is important to note that this protease inhibition mechanism differs from that of currently used FIP treatments such as molnupiravir or GS-441524, which primarily target the viral RNA-dependent RNA polymerase (RdRp), and are therefore not appropriate controls for a protease inhibition assay [32,33]. The absence of GC376, a highly specific FIPV M^pro^ inhibitor [34,35], was due to its unavailability in our laboratory at the time of the study.

The experimental results revealed that *P. indusiatus* at a concentration of 10 µg/mL achieved the highest inhibition of FIPV M^pro^ activity among the tested mushroom extracts, with an inhibition rate of 69.2% (Figure 3). Despite the mushroom extract’s inhibition rate being marginally lower than ritonavir and lopinavir, its efficacy as a natural extract warrants further investigation, suggesting the presence of bioactive molecules with FIPV M^pro^ inhibitory properties. This finding is particularly relevant because, while current gold standard FIP treatments focus on RdRp, viral proteases (specifically M^pro^ or 3CL^pro^) represent an equally critical and well-validated drug target for coronaviruses due to their essential role in processing polyproteins translated from viral RNA [35,36,37]. This enzyme is indispensable for viral polyprotein processing, which is a fundamental step for replication [37,38]. The success of protease inhibitors in other viral infections (e.g., Human HIV and Hepatitis C virus [HCV]), along with the promising results of FIPV-specific protease inhibitors such as GC376, underscores the druggability of this enzyme [39]. Also, due to the high conservation of M^pro^ during viral mutagenesis and its critical role in the coronavirus life cycle, M^pro^ is considered as a pivotal target for antivirus drug development [16]

Mushroom extracts are widely recognized for their antiviral and anti-inflammatory properties [40,41]. Previous studies have shown that polysaccharides and adenosine from crude mushroom extract inhibited SARS-CoV protein expression, genomic RNA synthesis, and spike protein activity [42,43,44]. El-Mekkawy, Sahar et al. (1998) have reported that triterpenes extracted from the fruiting bodies of *Ganoderma lucidum* can inhibit the essential protease enzyme of HIV-1 at a concentration of 7.8 µg/mL [45]. The findings of this study are consistent with previous reports. The crude extract from *P. indusiatus* is particularly noteworthy for further investigation of its anti-inflammatory properties, as it demonstrated the highest enzyme inhibition among the tested mushroom species and also showed potential for inhibiting the FIPV M^pro^. Exploring protease inhibitors from diverse natural sources, such as mushroom extracts, represents a vital strategy—offering novel compounds, alternative mechanisms to combat resistance, and a foundation for future combination therapies. Therefore, in this study, the crude extract from *P. indusiatus* was selected for testing its anti-inflammatory effects on PBMCs of donor cats and FIP fluid.

The PBMCs isolated from both the whole blood of donor cats and FIP fluid, characterized by monocytes and macrophages, are essential components of the innate immune system [46,47,48]. The selection of samples should prioritize PBMCs that are already isolated, do not clump, and exhibit a round, glossy appearance (Figure 4). The HIV/AIDS Network Coordination Standard Operating Procedure (HANC-SOP) recommends limiting the processing time to a maximum of 8 h [49]. Delays exceeding 24 h have been associated with decreased cell viability [22]. However, in this study, PBMCs were successfully separated from both whole blood and FIP fluid and tested within 24 h (Figure 5).

In the present study, we employed LPS to activate PBMCs as a model of inflammation. LPS, commonly referred to as endotoxin, is a potent pathogen-associated molecular pattern (PAMP) derived from the outer membrane of Gram-negative bacteria. It is recognized primarily by Toll-like receptor 4 (TLR4) expressed on the surface of immune cells, including monocytes/macrophages [50,51]. Engagement of TLR4 by LPS initiates downstream signaling cascades, notably the myeloid differentiation primary response 88 (MyD88)-dependent pathway, culminating in the activation of key transcription factors such as NF-κB and activator protein 1 (AP-1) [9,50,52,53]. Although FIPV is not a bacterial pathogen, viruses similarly trigger innate immune responses by expressing PAMPs, such as double-stranded RNA (dsRNA) and specific viral proteins [54]. These are recognized by pattern recognition receptors (PRRs), including endosomal Toll-like receptors (TLRs), such as TLR3, TLR7, TLR8, and TLR9, as well as cytosolic sensors like retinoic acid-inducible gene I (RIG-I) and melanoma differentiation-associated protein 5 (MDA5) [55,56]. Activation of these receptors initiates similar signaling pathways that converge on the activation of transcription factors NF-κB and AP-1 [10,11] (Figure 1). Although LPS was used as an inflammatory stimulant in this study, mimicking viral infection in feline PBMCs remains challenging due to the lack of a suitable viral culture system in our lab. Nevertheless, both LPS (via TLR4) and viral components (via other TLRs or cytosolic receptors) activate key pro-inflammatory pathways, particularly NF-κB and AP-1. This results in the upregulation of mediators such as TNF-α, IL-1β, IL-6, and iNOS, supporting the use of LPS as a relevant initial model for evaluating general anti-inflammatory effects.

The innate immune system’s ability to initiate responses to LPS was also examined [57]. In this report, 0.001, 0.01, 0.1, and 1 µg/mL of LPS were used to stimulate PBMCs isolated from whole blood of donor cats, compared with the absence of LPS (Figure 6). The data presented in this publication indicate that LPS increased cell viability by approximately 98–99% within 24 h, correlating with the report by Jansky et al. (2003) [58]. A study reported that LPS stimulation increased PBMCs proliferation by activating monocytes and T lymphocytes, thereby promoting their proliferation [59,60,61]. Based on the study results, LPS may have the potential to increase the number of PBMCs [59,60,61]. However, PBMCs isolated from FIP fluid do not exhibit LPS-induced inflammatory responses, likely because virus-infected cats already present with systemic leukocyte activation [23]. This diminished responsiveness may be attributed to the fact that FIPV specifically targets monocytes and macrophages, leading to their functional dysregulation [62,63].

The effect of *P. indusiatus* on nitrite scavenging activity in LPS-stimulated PBMCs was assessed. During the inflammatory response, nitric oxide production, measured as nitrite levels in the PBMCs’ supernatant, can be upregulated in freshly isolated PBMCs by in vitro stimulation with LPS [64,65,66,67,68]. LPS triggers the TLR4 pathway, leading to the activation of NF-κB and the upregulation of iNOS [50,51]. In LPS-induced samples, monocytes/macrophages are the primary NO producers (Figure 7A) [50,52]. In this study, a group of PBMCs, isolated from donor cat whole blood and stimulated with 1 µg/mL LPS, was evaluated for its anti-inflammatory effects, with a reduction in nitrite serving as an indicator [68]. The crude extract of *P. indusiatus* may possess potential anti-inflammatory properties (Figure 7B).

The test on the reduction of nitrite production by PBMCs isolated from FIP fluid (Figure 7D) clearly demonstrates that cats suffering from this disease naturally exhibit PBMC inflammation, even in the absence of LPS stimulation. The PRRs, such as TLR3, TLR7, RIG-I, and MDA5, lead to IFN signaling [69]. This may trigger IFN responses (IFN-α, IFN-β, IFN-γ), which can either promote NO production or have other immune effects [54]. The host’s recognition of viral DNA initiates multiple signal transduction pathways that converge on iNOS upregulation, leading to increased endogenous NO production [70]. The oxidative degradation of NO results in the formation of its breakdown products, nitrate and nitrite. This process, primarily identified in macrophages, involves the expression of the inducible iNOS enzyme [71,72]. Consequently, nitrite levels are significantly higher in PBMCs isolated from FIP fluid compared to those from the whole blood of healthy cats, with a statistically significant difference. This aligns with the research conducted by Özbek et al. (2022), which investigated serum nitric oxide levels in cats affected by FIP [73]. This elevation in nitrite serves as a reliable indicator of inflammation (Figure 7D) [74,75].

The results of this study show a statistically significant difference in the response to treatments between PBMCs isolated from whole blood and those from FIP fluid. PBMCs from whole blood responded to both treatments (dexamethasone and *P. indusiatus* extract) by reducing nitrite production, indicating potential anti-inflammatory effects. However, PBMCs from FIP fluid (possibly from an inflamed environment) did not respond in the same way (Figure 7C,D). The lack of anti-inflammatory effects from both the mushroom extract and dexamethasone in PBMCs from FIP-affected cats underscores the complex and severe nature of FIP-associated inflammation [23,54,62,63,69]. Unlike the acute response induced by LPS, FIP involves chronic, systemic inflammation driven by persistent viral replication in macrophages and a dysregulated immune response, characterized by persistently elevated levels of various inflammatory mediators (a systemic cytokine storm) [52,57,76,77]. In this hyper-inflammatory state, NO production may be maximally upregulated and “locked in”, reducing responsiveness to conventional treatments, including dexamethasone [54,55,56]. This suggests that FIP-related inflammation may involve distinct or more resistant pathways, as well as nitric oxide production through multiple mechanisms, compared to LPS models [10,11,54,56]. Additionally, the direct presence PBMCs from FIP fluid may alter cellular signaling compared to LPS-stimulated healthy PBMCs, potentially affecting drug responsiveness. The previous study suggests that dexamethasone has direct anti-inflammatory effects, acting by altering gene transcription—upregulating anti-inflammatory genes (e.g., IL-10), downregulating pro-inflammatory ones (e.g., IL-6, TNF-α, COX-2, iNOS), and inhibiting the NF-κB pathway [78,79]. In contrast, *P. indusiatus* modulates immunity and oxidative stress through compounds like β-glucans, which stimulate TNF-α, IL-6, and IL-10 to restore immune balance [7,80]. While dexamethasone suppresses NF-κB, *P. indusiatus* fine-tunes it to regulate immune responses [81,82].

Limitations of this study include, firstly, the use of LPS as a model of a general inflammatory stimulus instead of direct FIPV infection. Although both can activate similar inflammatory pathways (e.g., NF-κB leading to TNF-α, IL-1β, IL-6, and iNOS expression). FIPV infection is more complex, involving intricate virus–host interactions that LPS cannot fully mimic. Secondly, attempts to develop an in vitro FIPV infection model were unsuccessful. Although efforts were made to establish an FIPV infection model by infecting CRFK (Crandell-Rees Feline Kidney) cells in vitro, these attempts failed due to challenges in isolating the virus from FIP fluid samples, difficulties in quantifying viral load, and the inconsistency of infection. As a result of these limitations, it was not possible to use the actual virus in the study, making it necessary to rely on the LPS-based simulation system, which may not fully reflect the true nature of FIPV infection.

Future investigations may consider employing alternative approaches to better mimic the natural course of FIPV infection. For example, pseudotyped viral systems expressing the FIPV spike protein could provide a safe and controllable platform for examining viral entry and replication, while FIPV-infected cell lines may offer a more physiologically relevant model for studying virus–host interactions [83,84]. Although these models are not without limitations, they could complement the LPS-based stimulation system used in this study and thereby provide a more comprehensive evaluation of the antiviral and anti-inflammatory potential of the tested extracts.

Despite this, this study represents the first preliminary investigation to evaluate extracts from the mushroom *P. indusiatus* for both anti-FIPV M^pro^ activity and anti-inflammatory effects. It also represents the first assessment of the extract’s effects on PBMCs isolated from FIP fluid compared to PBMCs from healthy donors. The extract demonstrated strong inhibitory effects on FIPV M^pro^. Based on these results, this study is considered successful and has provided important and useful information, opening opportunities for developing new compounds, alternative mechanisms to overcome resistance, and serving as a foundation for future combination therapy research.

Future research should focus on elucidating the roles of pro-inflammatory cytokines (e.g., IL-6, TNF-α) and other relevant inflammatory mediators involved in the pathogenesis of FIP. Bioactive constituents from *P. indusiatus* should be investigated, and their chemical structures elucidated using advanced analytical techniques (e.g., liquid chromatography–mass spectrometry; LC-MS/MS). Subsequent evaluations should include both in vitro and in vivo studies to determine the pharmacological properties of the isolated compounds. Initial in vitro assays should assess antiviral efficacy and anti-inflammatory activity in relevant cell-based models. Promising compounds should then undergo in vivo validation using appropriate animal models. Additionally, comparative analyses with currently available FIPV-specific therapeutics are recommended to benchmark their relative efficacy and therapeutic potential.

## 5. Conclusions

This study provides compelling preliminary evidence for the dual antiviral and anti-inflammatory potential of *P. indusiatus* extract in the context of FIP. The extract showed a strong inhibitory effect (69.2%) on the FIPV M^pro^, comparable to established protease inhibitors such as ritonavir and lopinavir. It also significantly reduced NO production in LPS-stimulated PBMCs from healthy cats, indicating potent anti-inflammatory activity. Importantly, the extract was also tested on PBMCs isolated from FIP fluid, which are characterized by chronic inflammation and immune dysregulation. However, these cells showed limited responsiveness (nonresponse) to both dexamethasone and *P. indusiatus* extract, underscoring the complexity of immune activation in FIP. Although LPS was used as a surrogate inflammatory stimulus and a direct FIPV infection model was not available, this is the first study to evaluate *P. indusiatus* for both antiviral and anti-inflammatory effects in the context of FIP. These findings highlight the potential of *P. indusiatus* as a natural source of immunomodulatory agents. Future research should focus on isolating its active compounds, elucidating their mechanisms in relevant infection models, and evaluating their therapeutic potential in vivo, including direct comparisons with FIPV-specific inhibitors.

## Data Availability

The raw data supporting the conclusions of this article will be made available by the authors on request. Further inquiries can be directed to the corresponding author.

## Supplementary Materials

The following supporting information can be downloaded at https://www.mdpi.com/article/10.3390/vetsci12090847/s1, Figure S1: The appropriate concentration of DMSO for the viability of PBMCs., and Table S1: Optimization of *P. indusiatus* PBMC viability.
