# Supplementary figures and images for: Evaluation of the Dual Antiviral and Immunomodulatory Effects of Phallus indusiatus in a Feline Infectious Peritonitis Model Using PBMCs
Source: Vet Sci. 2025 Sep 1;12(9):847. doi: 10.3390/vetsci12090847 (PMC12474325; doi:10.3390/vetsci12090847)

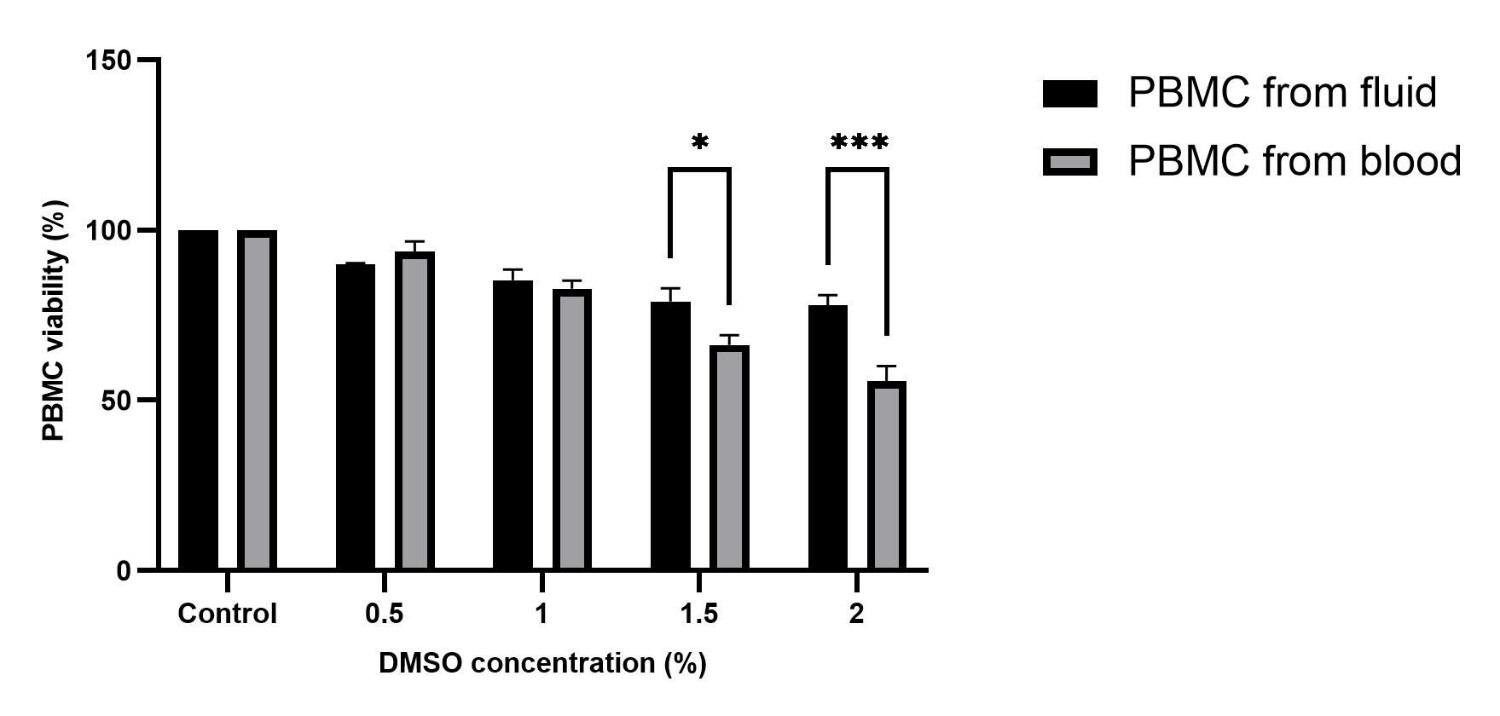

Supplement: Supplementary file 1 [file vetsci-12-00847-s001.zip › FigureS1.jpg]
