# Supplementary material for: Evaluation of the Dual Antiviral and Immunomodulatory Effects of Phallus indusiatus in a Feline Infectious Peritonitis Model Using PBMCs
Source: Vet Sci. 2025 Sep 1;12(9):847. doi: 10.3390/vetsci12090847 (PMC12474325; doi:10.3390/vetsci12090847)
Supplement: Supplementary file 1 [file vetsci-12-00847-s001.zip › TableS1. .doc]

TableS1. Optimization of *P. indusiatus* PBMCs Viability.

| *P. indusiatus* concentration (µg/mL) | PBMCs viability (%) |
| --- | --- |
| 10,000 | 25.16 ± 9.56 |
| 5,000 | 37.14 ± 10.54 |
| 2,500 | 47.76 ± 12.56 |
| 1,250 | 59.44 ± 13.66 |
| 525 | 61.14 ± 13.66 |
| 312 | 70.59 ± 17.52 |
| 156 | 81.71 ± 4.93 |
| 39 | 85.29 ± 2.96 |
| 19 | 93.22 ± 8.32 |
| Control | 100 |
